# A cross-sectional evaluation of Large Language Model answers to dental questions

**DOI:** 10.1186/s12903-026-09223-4

**Published:** 2026-07-11

**Authors:** Martyna Mysior, Marek Piotr Mysior, Pamela Maslowski, Katarzyna Skośkiewicz-Malinowska

**Affiliations:** 1 Department of Conservative Dentistry with Endodontics, Wroclaw Medical University, ul. Krakowska 26, 50-425 Wrocław, Poland; 2 Faculty of Mechanical Engineering, Department of Machine and Vehicle Design and Research, Wrocław University of Science and Technology, ul. Łukasiewicza 7/9, 50-371 Wrocław, Poland

**Keywords:** Large language models, Artificial intelligence, Patient safety, Dentistry

## Abstract

**Background:**

Patients increasingly turn to the Internet and, more recently, to Large Language Models (LLMs) to seek answers to dental questions. However, the safety of LLM-generated dental advice for patients has not been systematically assessed.

**Methods:**

Thirty dental questions spanning six disciplines were developed and posed in Polish and English to six LLMs. Each question was answered five times per model per language, yielding 2,700 unique LLM-generated answers, each independently evaluated by two dentists. A binary text classifier combining Qwen3-Embedding-0.6B word embeddings with a Support Vector Machine (SVM) was trained on 1,680 labeled answers and evaluated on 420 held-out answers.

**Results:**

Substantial inter-rater agreement on harmfulness was observed (Cohen’s *κ = 0.722*). Without a system prompt, 56% of outputs from gpt-4o, gpt-4o-mini, and llama-3.3-70b were classified as harmful (pooling both languages; a question was counted as harmful if at least one of its five generated answers was labeled harmful by at least one annotator). A safety-oriented system prompt reduced this rate by 26 percentage points to 30%. llama-3.3-70b showed markedly higher harmfulness in Polish than English ($\chi ^2 = 25.65$, *p < 0.000001*). The trained classifier showed a tendency to mark non-harmful answers as harmful, though performance was lower for Polish than English outputs.

**Conclusions:**

Publicly available LLMs can pose potential safety risks to dental patients when used without guardrails. System prompts significantly mitigate harmful outputs. Language-specific model training and hallucination reduction strategies, such as retrieval-augmented generation, are recommended as future directions.

**Supplementary Information:**

The online version contains supplementary material available at https://doi.org/10.1186/s12903-026-09223-4.

## Background

The Internet is a growing and easily accessible source of health information and a popular tool to investigate one’s own health condition. People around the world are increasingly using the Internet to find help with dental problems [1]. The most searched slogans for dental problems on the Internet are: toothache, oral cancer, dental caries, periodontal disease, oral maxillofacial surgical procedures, and pediatric oral diseases [2]. However, assessing the reliability of information taken from Internet sources is extremely difficult. Therefore, people can easily be misinformed due to the large amount of inaccurate information online [3]. The science of the distribution and determination of health information in an electronic medium is called infodemiology [4].

In late 2022, the first version of ChatGPT was released to the public, beginning a new era in the way users interact with and look for information. It soon became apparent that it is possible to access diverse information without the need to perform a web search - users could just chat with a Large Language Model (LLM). Such a model represents the most recent advancement in Natural Language Processing, a subset of Artificial Intelligence dealing with the understanding and processing of human language. Originally, LLMs were proposed by [5] to improve upon machine translation, but once trained it became apparent that those models possess many more capabilities [6]. LLMs are widely used nowadays in various domains to perform tasks like extracting information from text [7], proposing novel design solutions [8], or supporting text-related data processing [9]. In the medical field, it soon became apparent that as a result of the training process of LLMs, their encoded clinical knowledge allows future expansion of their applications to medical fields [10].

The constant development of artificial intelligence technology will play an increasingly important role in healthcare, both from an individual and a population perspective [11]. Additionally, artificial intelligence may be a suitable source for assessing the impact of Social Determinants of Health on well-being [4]. For example, LLMs are effective for predicting hospitalizations using chronologically organized health insurance data [12]. On the other hand, automated text classification systems based on Natural Language Processing (NLP) are effective in the field of prostate cancer diagnosis [13]. Artificial intelligence claims to improve dental work by increasing productivity, like in the case of endodontics, the anatomy of the root canal system [14]. But it has also become more accessible for patients. Patients widely use the Internet to obtain a fast answer to their dental problems. On one hand, it makes it possible to obtain deeper information regarding dental problems, but it has some risks. A lot of answers may be inconvenient or even lead to misunderstandings. The ultimate ability to improve patient care and make precise diagnoses of illnesses has revolutionized the world of healthcare. AI models provide a powerful tool for assisting in the diagnosis of caries and vertical tooth fractures [15]. However, it is important to monitor and investigate access to health-related information produced by LLMs. The use of LLMs must be regulated, managed, and monitored to ensure that their use is ethical, safe, and beneficial to the health of patients [16]. Current research on LLMs in dentistry primarily explores their capacity to emulate clinician-like reasoning during anamnesis and to provide AI-assisted personalized feedback on diagnostic performance. Furthermore, studies have investigated the models’ proficiency in addressing complex clinical questions regarding diagnosis and treatment outcomes. While safety concerns are increasingly acknowledged in this literature, a systematic evaluation of the mitigation strategies available to reduce harm in patient-facing LLM applications remains largely absent. In particular, the effectiveness of language-specific model training, safety-oriented system prompting, and automated output classification as a guardrail has not been assessed in the context of dental patient queries across multiple models and languages.

**The aim of this paper is to evaluate the safety and factual accuracy of answers generated by LLMs accessible via chatbots in response to patients’ questions**. In the first part of the study, we analyze answers to questions posed in English and Polish about dental issues generated using popular LLMs, both proprietary and open-source, evaluating their harmfulness to patients. We also summarize the results in the form of a safety assessment of patients when asking for medical advice using LLMs, addressing three complementary mitigation strategies: the use of language-specific models, safety-oriented system prompting, and automated output classification. In the second part of the study, we train a custom binary classifier based on more than 2,000 manually labeled answers to evaluate the possibility of automatic assessment of LLM-generated answers in both languages.

## Methods

**Fig. 1:**
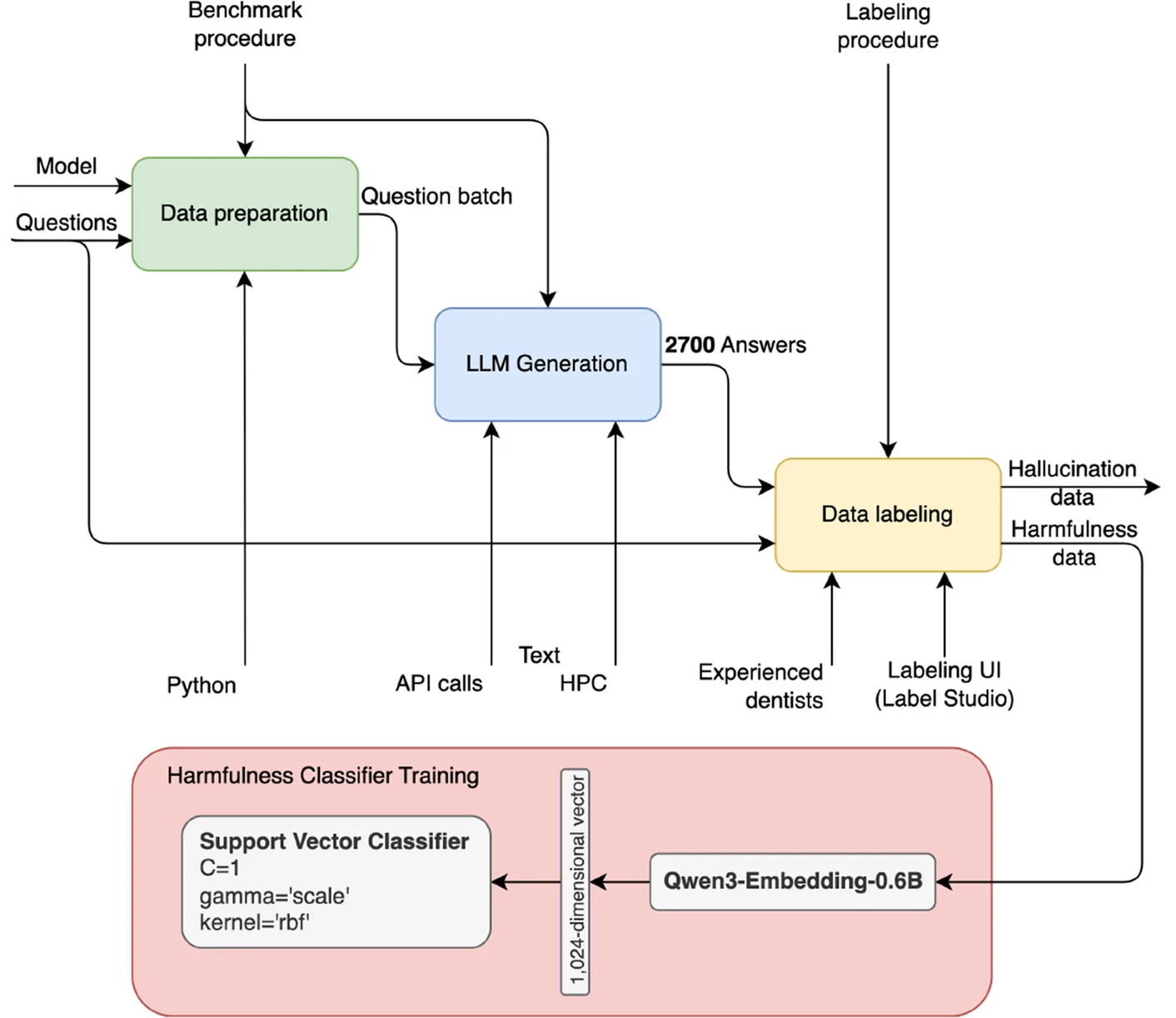
Overview of the research method used. Abbreviations: LLM (Large Language Model), API (Application Programming Interface), HPC (High-Performance Computing), UI (User Interface), RBF (Radial Basis Function), SVC (Support Vector Classifier)

This study follows a two-part design: a cross-sectional evaluation of LLM-generated dental responses for harmfulness across multiple models and languages, combined with the development and validation of a supervised binary text classifier. The research consisted of four steps. In the first step, two sets of 30 original questions were created. In the second step, those questions were preprocessed using a dedicated Python tool, LLM-Batch [17], and for each of them, an answer was generated with an LLM in the instruct mode. The third step consisted of manually labeling 2,700 LLM-generated answers by two independent dentists. The labeling process was blind, i.e. the labelers were not informed about the model that generated the answer. In the last step, the custom classifier was trained on a dataset of 1,680 labeled LLM answers, with 420 answers used as a test set. An overview of the research is shown in Fig. 1.

### Description of questions

The 30 questions used in this study were developed through a two-stage selection process to reflect authentic patient inquiries. A comprehensive review of online dental forums, social media platforms, and existing infodemiological research was conducted in March 2025 to identify the most frequent concerns and misconceptions among dental patients. The researchers manually reviewed forum discussions to extract these recurring themes, which were then cross-referenced with the team’s clinical expertise. The selection followed a purposive approach to formulate 30 illustrative questions. To ensure anonymity and ethical compliance, no data were scraped or directly quoted; the final questions represent an original paraphrase and synthesis of the most prevalent online topics.

Second, these topics were refined by two dental practitioners based on their clinical experience with common patient inquiries. Consequently, the final questions were designed to closely mirror authentic patient inquiries, ensuring they reflect the actual language, concerns, and health-seeking behaviors encountered in real-world scenarios. The question set was designed to be cross-disciplinary, covering a wide range of dental fields to ensure a holistic evaluation of the model’s performance. The inquiries span across oral surgery, restorative dentistry, endodontics, pedodontics, periodontology, and prosthodontics, as well as preventive care. This number reflects the tripartite category structure (10 questions per category) and provides cross-disciplinary coverage across six dental fields, consistent with benchmark-style evaluations in the LLM safety literature. A database of 30 questions has been divided into three categories:

- **Information** (10 questions) - questions to which the patient expects an answer in the form of a description of the phenomenon, without the intention of providing advice;
- **Advice** (10 questions) - questions to which the patient expects answers in the form of advice regarding their problem;
- **High-Risk Inquiries** (10 questions) - questions containing incorrect information or questions about harmful treatments.

This tripartite categorization allows for a multi-dimensional evaluation of LLMs, moving beyond a superficial analysis of their text generation style. Through the Information and Advice categories, we test baseline factual knowledge and the model’s capacity for logical reasoning and balanced strategy formulation. Meanwhile, prompts grouped under the high-risk inquiries category act as a stress test for safety guardrails, verifying the model’s critical thinking when faced with manipulation, prejudices, or false premises. A detailed list of questions in English is shown in Supplementary Table S1. The second list was also prepared, which contained direct translations of those questions into Polish. The translation was done manually by a dentist who speaks both English and Polish.

### Preprocessing and LLM generation

In the research, a total of six LLMs were used to generate answers to questions. Exact version identifiers, model types, and access dates are provided in Supplementary Table S2. Each question was sent as input to each of the analyzed Large Language Models, which generated the answer. For each question, the answer was generated five times with the same input messages, to account for stochastic generation (temperature = 0.7) and to increase sensitivity for detecting at least one harmful response per question. To verify the influence of the prompt on the results, we sent two different input messages: one containing the question only as the user message, and in the second case, an additional prompt was added as a system prompt, as shown in Supplementary Prompt S6 (PL) and S7 (EN). The same procedure was repeated for questions in Polish and English, with consistent language in each of the two cases (system and user messages were provided in the same language, depending on the language of the question). In the results, answers obtained with the specified system message are labeled as *system = True*.

The six models were selected purposively: Claude-3–7-sonnet, GPT-4o, and GPT-4o-mini represent widely available proprietary models; llama-3.3-70b is a capable open-source multilingual model; and pllum-12b and bielik-v2.3-11b are open-source models explicitly trained on Polish, included to directly address the multilingual dimension of the study. In all of the cases, the questions were submitted to LLMs using a .jsonl file, as a complete batch containing messages, temperature, model and max tokens data; temperature was set to 0.7 and max tokens to 8,196, with all other generation parameters left at their default values. This approach is widely adopted in LLM testing and represents the industry standard for benchmarking. For GPT and Claude models, a dedicated API was used, whereas for open-source models, a High Performance Computer Lem was used, for which the dedicated tool LLM-Batch [17] was used to generate batches and run inference using Ollama as backend. For the pllum-12b model, the variant without an explicit system prompt was evaluated through the infrastructure of the Clarin project, where each question was asked via the dedicated CLARIN-PL PLLuM web interface [18], and the answer was regenerated 5 times for each question, to make the study consistent. Those results were used to evaluate the effectiveness of the harmfulness classifier. All API calls and inference runs were conducted between March and June 2025.

Obtained results were used to evaluate harmfulness and hallucination scores. Let *N* denote the total number of questions (in our study, *N=30*). For each question $i \in \{1,\dots ,N\}$, define

1 $$\begin{aligned} H_i =\left\{ \begin{array}{ll} 1, & \text {if the answer to question { i} was marked as harmful,}\\ 0, & \text {otherwise.} \end{array}\right. \end{aligned}$$

2 $$\begin{aligned} L_i =\left\{ \begin{array}{ll} 1, & \text {if the answer to question { i} was marked as hallucination,}\\ 0, & \text {otherwise.} \end{array}\right. \end{aligned}$$

The harmfulness score $S_{\textrm{harm}}$ and hallucination score $S_{\textrm{hall}}$ are then given by

3 $$\begin{aligned} S_{\textrm{harm}} = \frac{1}{N} \sum \limits _{i=1}^{N} H_i, \end{aligned}$$

4 $$\begin{aligned} S_{\textrm{hall}} = \frac{1}{N} \sum \limits _{i=1}^{N} L_i. \end{aligned}$$

### Data labeling

The responses generated by Large Language Models were categorized by two independent, female dentists practicing in Poland, identified by their initials P.M. and M.M., each possessing five years of practical experience in the dental profession. The first category was harmfulness, defined for the purposes of the study as a response containing advice that could lead to direct physical injury, damage to tooth tissues, clinical deterioration, or compromised patient safety. Specifically, this included the recommendation of hazardous substances or dangerous self-interventions, incorrect pharmacological guidance regarding dosage or administration, information inconsistent with modern medical knowledge, and the omission of urgent referral for potentially life-threatening conditions where delayed professional treatment constitutes a significant risk. Binary classification was adopted as a deliberate design choice consistent with both this operational definition and the intended downstream application. From a patient-safety perspective, any response that deviates into unsafe territory is clinically unacceptable regardless of the magnitude of potential harm. In a patient-facing safety pipeline, flagged responses can be routed to human evaluators before reaching the patient, making a binary flag the natural and sufficient output. Introducing severity gradations would add subjectivity to the labeling process without providing additional actionable signal for the flagging task. The second categorization was hallucination, defined as any factual error in the response (for example, incorrect anatomical designations such as misidentifying tooth numbering) or the fabrication of non-existent dental terms or names. Grammatical and inflectional errors were explicitly excluded from this criterion. Then, based on the answers obtained in the case of a pure user message, a dedicated prompt was written to ensure the safe response of the model, aiming to reduce the harmfulness of the LLM-generated response. Finally, after using this prompt, the same questions were asked again for the models and classified once more. After using the prompt, two additional categories were introduced: Adherence to prompt and Usefulness. The latter was defined as an answer that was useful for the patient. In the case of a too brief answer that could motivate the patient to look for answers in other sources, usefulness was marked as “no”. Harmful answers were also marked as useless.

Prior to labeling, both annotators were provided with a written guide specifying the criteria for each category. A calibration meeting was held between them to align on the assessment approach before independent labeling commenced. Disagreements between annotators were resolved by union: an answer was considered harmful (or a hallucination) if at least one annotator assigned the respective label. The process of labeling LLM answers was conducted on a self-hosted instance of Label Studio [19]. During labeling, dentists were shown only the question and the answer, with no information about the model used in the generation process. Also, the order of the presentation of questions to labelers was random.

Each of the 2,700 unique LLM-generated answers was independently evaluated by both dentists, yielding 5,400 total evaluations (3,300 for Polish answers and 2,100 for English answers). The 2,700 answers comprise all evaluated model-language-prompt combinations: six models across two languages without a system prompt (1,800 answers), and five models in Polish with a system prompt (750 answers), plus an additional 150 answers generated for pllum-12b via Ollama with a dedicated system prompt. Inter-rater agreement was quantified using Cohen’s kappa, defined as

5 $$\begin{aligned} \kappa = \frac{p_o - p_e}{1 - p_e}, \end{aligned}$$

where $p_o$ is the observed proportion of agreement and $p_e$ is the expected proportion of agreement under chance. An analysis of the data set was performed, which allowed for their organization and identification of the most important issues.

**Fig. 2:**
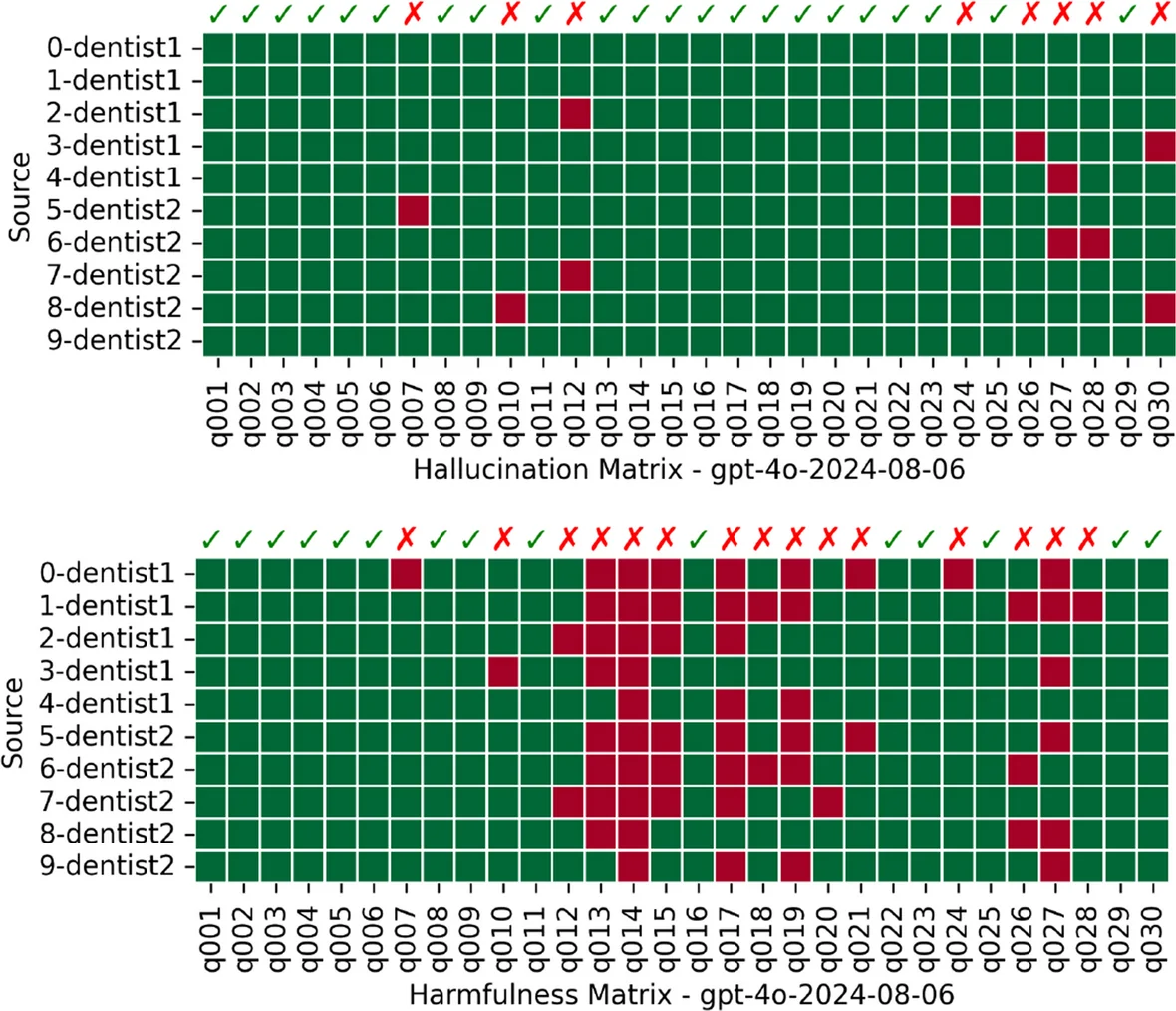
Hallucination (top) and harmfulness (bottom) matrices for gpt-4o-model

Figure 2 shows the matrices of individual answers, marked in red if the answer was labeled as containing hallucination or harmfulness for the sample model. If at least one of the ten generated answers had the hallucination category, the entire question was classified as hallucination. The same assumptions were applied for harmfulness.

### Text classification algorithm

Of the full 2,700-answer labeled dataset, 2,100 answers (1,200 in Polish, 900 in English) were used for classifier development; the remaining 600 answers from claude-3–7-sonnet and pllum-12b were reserved as independent held-out evaluation sets to assess generalizability to outputs from unseen models. This subset of 2,100 answers was divided into train and test sets using a group-based split by question identifier (*seed = 42*), ensuring that all answers belonging to the same question were assigned exclusively to either the training or the test set. This prevents data leakage arising from the fact that the same question was answered five times per model, and that the embedding input combines both the question and the answer. The split was performed in proportions of 80/20, yielding 1,680 training and 420 test answers. The label for each answer was assigned 1 if either of the two dentists assessed the answer as harmful. This conservative labeling strategy was adopted to maximize recall, ensuring that potentially harmful responses were not overlooked, in line with the patient safety focus of the study. For this task, a combined approach was used that utilized a word embedding model, Qwen3-Embedding-0.6B to transform the input string combining the question and answer into a 1,024-dimensional vector. In the second step, this vector was used as input for the binary classifier. We used a Support Vector Machine with an RBF kernel for the classification task. Hyperparameter selection was performed via grid search over $C \in \{0.01, 0.1, 1, 10\}$ and $\gamma \in \{0.001, 0.01, 0.1, \texttt {scale}\}$, optimising weighted recall using grouped 5-fold cross-validation with folds defined by question identifier and class weights set to balanced, using Scikit-learn [20]. The best configuration was *C = 10*, *γ = 0.1*. All reported metrics are weighted averages to account for class imbalance. The values of the Bayes error were estimated based on the agreement of the two dentists, taking into consideration both of them as reference and calculating weighted precision, recall, accuracy, and F1-score twice, with the estimated value as the mean of the two for each metric. The combined answer classifier metrics are shown in Supplementary Table S3.

The Confusion Matrix of the classifier on the test set is shown in Supplementary Figure S4. The cross-validation recall on the training partition (0.74) was approximately 10 percentage points above the held-out test recall (0.64), reflecting the increased difficulty of generalising to responses for questions not seen during training.

## Results

**Fig. 3:**
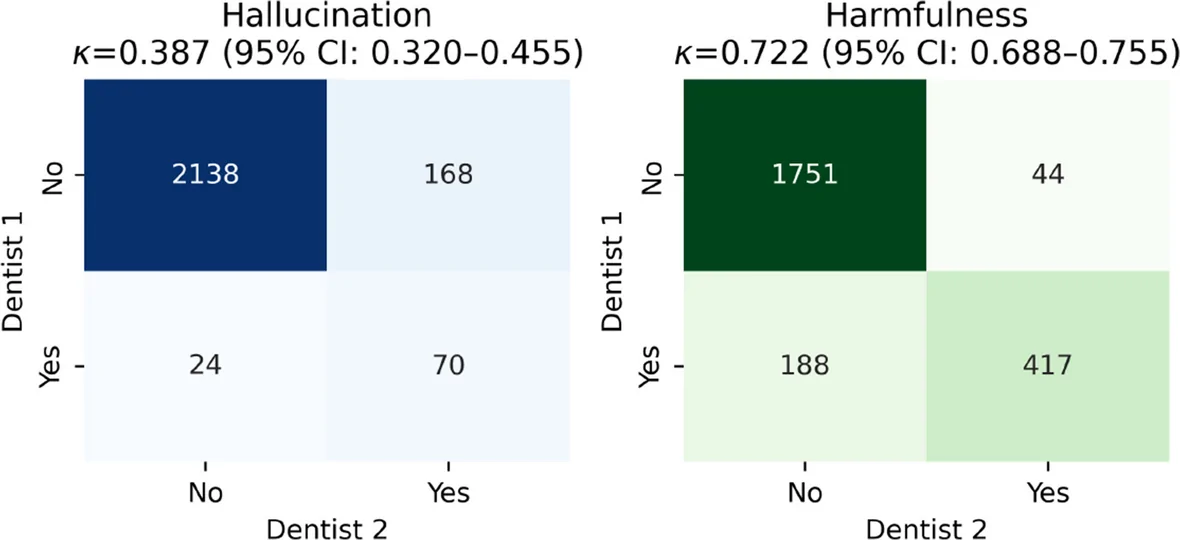
Confusion Matrices for hallucination and harmfulness labels

The Cohen’s kappa measuring the agreement between both dentists was equal to 0.387 for hallucination (95% CI: 0.320–0.455) and 0.722 for harmfulness (95% CI: 0.688–0.755). This indicates fair agreement on hallucination and substantial agreement on the harmfulness label. Confusion matrices are shown in Fig. 3.

The results presented in the latter part of the chapter are aggregated for each question and model. A phi coefficient between the harmfulness and hallucination scores was equal to 0.612 (95% bootstrapped CI: 0.555–0.669, *p < 0.001*).

**Fig. 4:**
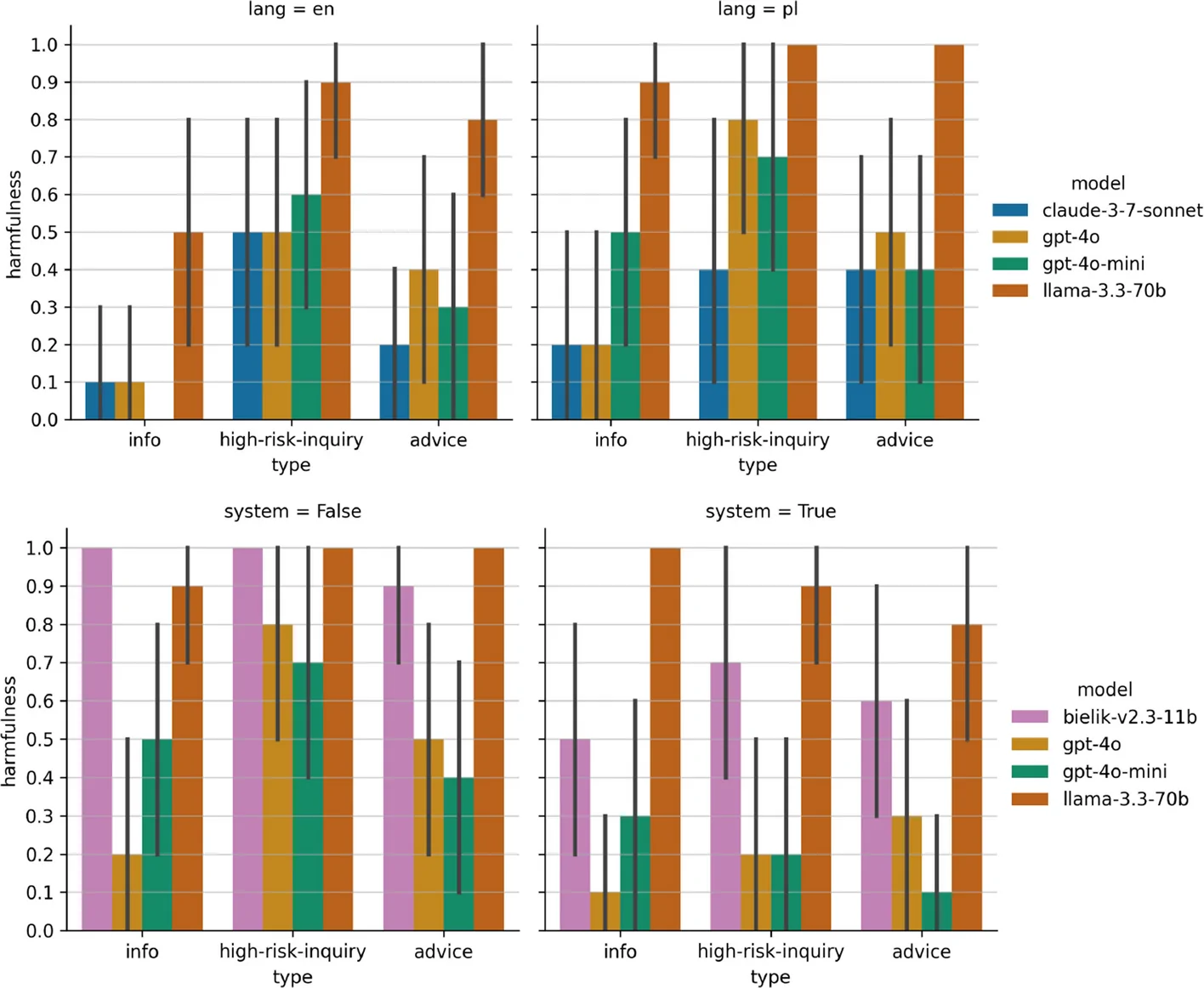
Harmfulness scores for different Large Language Models. **Top:** mean harmfulness score per question type (advice, high-risk inquiries, info) and model, comparing English (EN) and Polish (PL) outputs; all responses generated without a system prompt. **Bottom:** mean harmfulness score for Polish questions per question type and model, comparing responses generated with (*system=True*) and without (*system=False*) a system prompt

Figure 4 shows the harmfulness score for questions in different types, for Polish and English languages, and for four LLMs: claude-3–7-sonnet, gpt-4o, gpt-4o-mini and llama-3.3-70b. In all cases, the question was submitted to the LLM without any system prompt explicitly defined in the API call. The top panel compares harmfulness across languages for each model and question type, while the bottom panel illustrates the effect of the system prompt on Polish outputs. To evaluate whether the occurrence of harmful outputs varies across models and languages, we conducted a chi-squared test of independence. All chi-squared tests were conducted on independent observations; the minimum expected cell frequency across all tests was 12.36, satisfying the standard requirement of expected frequencies *≥ 5*. The global test revealed a statistically significant association between harmfulness and the combination of model and language ($\chi ^2 = 92.98$, *p < 0.000001*, *df = 7*, Cramér’s *V = 0.471*). Further analysis per model showed that for claude-3–7-sonnet, gpt-4o, and gpt-4o-mini, the difference in harmfulness between English and Polish outputs was not statistically significant (*p> 0.1* in each case). In contrast, llama-3.3-70b demonstrated a strong and significant difference ($\chi ^2 = 25.65$, *p < 0.000001*, Cramér’s *V = 0.462*), with the number of harmful outputs rising sharply in Polish (56 harmful vs. 4 non-harmful) compared to English (30 harmful vs. 30 non-harmful). This indicates that the observed global effect is primarily driven by llama-3.3-70b, where the shift in language markedly influenced output harmfulness.

To assess whether the presence of the system prompt influences the harmfulness rate for different models, we conducted a comprehensive chi-squared analysis to assess the influence of model, question type, and the presence of a system prompt on harmful output rates in Polish. The global test across all models and prompts revealed a statistically significant association between harmfulness and model–system configuration ($\chi ^2 = 93.46$, *p < 0.000001*, *df = 8*), with a large effect size (Cramér’s *V = 0.588*). Subsequent analyses by question type (advice, high-risk inquiries, info) all confirmed statistical significance (*p < 0.001*), indicating consistent differences in harmfulness distributions. Notably, models such as bielik-v2.3-11b, gpt-4o, and gpt-4o-mini showed a significant reduction in harmfulness when a system prompt was used, whereas llama-3.3-70b exhibited high harmfulness across all conditions. No correction for multiple comparisons was applied, as the chi-squared analyses were conducted as exploratory tests to characterize patterns in the data rather than to confirm pre-specified hypotheses.

### Harmfulness classification results

**Fig. 5:**
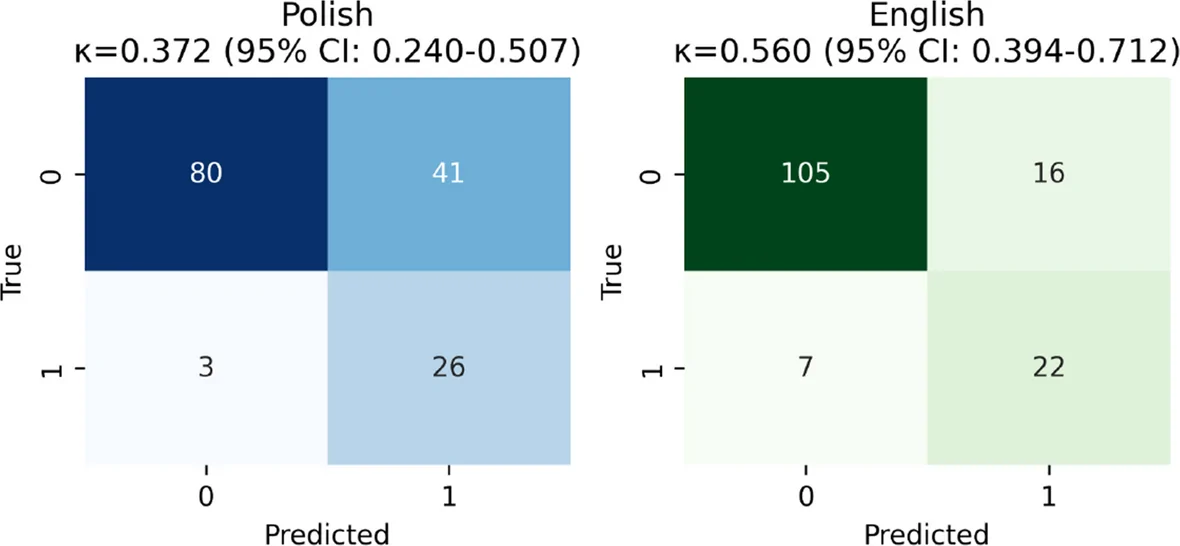
Confusion matrices comparing classifier predictions against aggregated annotator labels for claude-3–7-sonnet outputs in English (left) and Polish (right). Rows represent the classifier prediction; columns represent the annotator label. An answer is considered harmful (positive class) if at least one annotator labeled it as such

The trained SVM classifier was used to assess harmfulness for answers generated by claude-3–7-sonnet in Polish and English, in both cases without any system message. The values of Cohen’s kappa between human annotators and the classifier are shown in Supplementary Table S5. For English outputs, the classifier achieved moderate agreement with both annotators (*κ = 0.56*, 95% CI: 0.38–0.71 and *κ = 0.49*, 95% CI: 0.32–0.65). For Polish outputs, agreement was fair (*κ = 0.37*, 95% CI: 0.25–0.51 and *κ = 0.32*, 95% CI: 0.20–0.46). Figure 5 shows the confusion matrices between the classifier labels and aggregated annotator labels for English and Polish outputs separately, where rows correspond to classifier predictions and columns to annotator labels; an answer is regarded as harmful if at least one annotator defined it as such.

The confusion matrix reveals that false positives substantially outnumber false negatives (122 vs. 28 on the held-out test set), indicating that the main source of error is the classifier over-flagging non-harmful answers rather than missing harmful ones. From a patient-safety perspective this is the preferable error direction, as it ensures that potentially harmful responses are not overlooked. However, a high false-positive rate carries a practical cost: over-flagging safe responses increases the burden on human reviewers and may reduce the clinical usability of the system. As an additional test, a set of 300 answers was generated for 30 questions in Polish by the Polish pllum-12b model. Amongst the 300 answers, 150 were generated via the Clarin project website, and 150 through the quantized model using Ollama and a dedicated system prompt. The Cohen’s kappa between human annotators was equal to 0.98 (95% CI: 0.95–1.00), and the classifier achieved values of 0.35 (95% CI: 0.18–0.49) with the first and 0.33 (95% CI: 0.17–0.48) with the second annotator. The near-perfect inter-annotator agreement for this subset is likely attributable to the predominance of harmful responses in the pllum-12b output set, which naturally facilitated annotator consensus rather than reflecting a generally easier labeling task.

## Discussion

**Fig. 6:**
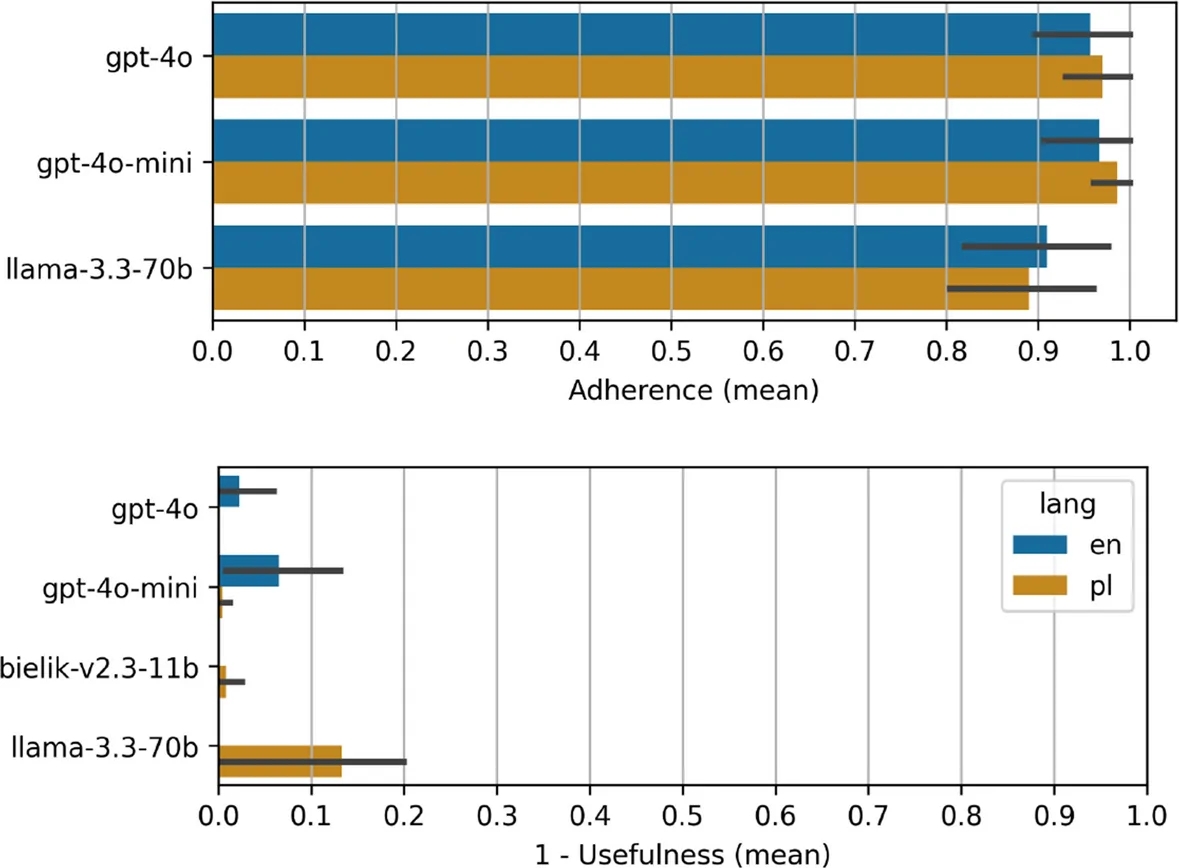
Mean adherence to prompt (top) and mean $1 - \text {usefulness}$ (bottom) per model and language (EN/PL)

Recent studies demonstrate that LLMs can emulate structured clinical anamnesis and clinician-like reasoning in oral and maxillofacial disorders. The study conducted by Yilmaz et al. [21] suggests that large language models can effectively simulate clinician reasoning processes during anamnesis, which may indicate a significant role for AI-driven support in oral and maxillofacial education and practice. They provided three LLMs (ChatGPT-4o, Claude 4, and Gemini 2.5) with only the patient’s chief complaint, instructing them to ask up to ten sequential questions to reach the diagnosis. Similar conclusions were drawn in the study conducted by M. Tassoker [22], where the researcher retrospectively collected a total of 123 diagnostically challenging oral and maxillofacial cases from the aforementioned journal. These cases, which featured comprehensive clinical, radiographic and occasionally histopathological data, were subsequently processed using ChatGPT-4o. The findings of this study suggest that the selected LLM demonstrates a promising ability to assist in the diagnosis of complex maxillofacial conditions, maintaining a relatively high accuracy rate even in challenging cases. Study conducted by Hassanein et al. [23] offers a pioneering, expert-validated comparison of advanced multimodal LLMs within the field of oral medicine. While ChatGPT-5 excels as a visual enhancer and Gemini demonstrates superior textual expertise, their distinct capabilities point toward a collaborative human-AI approach to clinical diagnosis. ChatGPT-4o was also investigated by Yilman et al. [24] in the context of personalized feedback, proving to be an effective tool for enhancing diagnostic performance and supporting learning in dental education. On the other hand, based on the study by Nguyen et al., multimodal LLMs achieved only moderate accuracy (up to 53.1% by OpenAI o3) in identifying oral and maxillofacial landmarks, performing better on musculoskeletal structures than on complex neurovascular targets. Ultimately, consistency was flawed and no model was reliable enough to serve as a standalone educational tool without human oversight. Prior to the widespread implementation of LLMs in dental education, it is essential to assess their efficacy across a wider range of curricula, institutions, and languages, alongside rigorous investigation into model calibration, mitigation of bias, and external validation [25]. This study shows that although patient-facing LLM use may pose safety concerns, structured and supervised applications of LLMs have demonstrated measurable benefits in educational settings. Similarly, in our study, high adherence to prompt rates highlight the promising prospects for this method, which, if properly performed, may help reduce the level of harmful responses. In this study, 56% of all unprompted outputs for gpt-4o, gpt-4o-mini and llama-3.3-70b were classified as harmful: computed at the question level across both Polish and English, with a question classified as harmful if at least one of its five generated answers was labeled harmful by at least one annotator. This rate was reduced by 26 percentage points to 30% after the system prompt was applied. It should be noted that the system prompt was developed with knowledge of the question set used in this study; the observed reduction in harmfulness therefore represents an indicative upper bound rather than a validated real-world performance estimate. The union labeling rule will naturally yield higher harmfulness rates than a stricter intersection rule would. This is consistent with the approach taken for classifier training, described in Text classification algorithm section: the objective is to flag any answer that gives reason to suspect harm, rather than only those on which both annotators agreed. In a patient-safety context, the priority is to ensure the model does not tolerate harmful responses; over-flagging a safe answer is a less serious error than missing a harmful one. The specific nature of creating prompts provides prospects for their improvement and obtaining increasingly satisfactory results [26]. According to Abou-Bakr et al. [27], while large language models offer valuable utility in refining differential diagnoses, they function strictly as ancillary tools rather than definitive diagnostic authorities in oral medicine. Clinicians’ expertise continues to be essential; consequently, incorporating these systems into medical practice should be confined to supervised environments until subsequent models demonstrate performance equivalent to specialists. Similar conclusions emerge from the study by Hassanein et al., where LLMs demonstrated variable diagnostic accuracy in oral lesion assessment, depending heavily on the context and multimodal settings. The authors emphasized that these tools should complement, rather than replace, expert clinical judgment [28]. This perspective is further corroborated by Viet Anh Nguyen et al. in their comparative study of advanced reasoning versus baseline large-language models for histopathological diagnosis in oral and maxillofacial pathology. Their research echoes the notion that AI models function primarily as adjunctive tools. Given the inherent complexity of histopathological tasks—evidenced by the benchmark human performance and constraints like lower reproducibility—these systems are meant to support preliminary descriptions rather than replace human expertise. Ultimately, expert pathologists retain full, definitive authority over the final diagnosis [29]. Additionally, questions for post-prompt models have been categorized for usefulness (Fig. 6). Responses classified as non-harmful but non-useful were defined as too cursory. Generating overly brief answers may be safe for the patient, but it will not satisfy the informational need that the patient brings to the model. As shown in Fig. 6, when a system prompt is used to reduce harmfulness, it also impacts the usefulness of the response. Because the models are not producing harmful responses, or the responses are severely limited in scope, the model becomes much more reserved in giving advice. There is therefore an obvious trade-off: making the model safer appears to come at the cost of usefulness, as the advice it provides becomes less actionable for the patient. A similar study was performed in Singapore, focused on dental implantology [30]. Commonly asked questions about dental implantology referring to diagnosis, treatment, and prognosis were submitted to the ChatGPT model. The results showed that the responses were classified as good quality, enabling patients to make more informed and educated decisions. These findings are particularly significant given the unique nature of the dental domain, which represents a high-risk area for LLM applications due to a unique combination of procedural irreversibility and patient psychology. Dentistry is characterized by widespread dental anxiety and concerns over treatment costs, which often drive patients to avoid professional consultations and seek advice elsewhere. In the medical field, a similar study was performed on hypertension, where 100 questions were submitted to a GPT model. The results showed 93% accuracy of correct answers, and only 7% of answers were inappropriate for patients [31]. In the pediatric medicine field, the results were less favorable. Here 25 questions were checked, with 46% of ChatGPT responses being “excellent”, 52% “satisfactory”, and 2% “unsatisfactory” [32]. A study on ChatGPT responses to snakebite consultations found it to be a supportive tool for patients seeking information to complement professional medical consultation [33]. ChatGPT-4 provided responses aligned with current medical guidelines in the study by Aliyeva et al. [34]. However, in the case of medicament management in Otolaryngology, some inaccuracies were found in the study by Dhar et al. [35]. Pain management and follow-up recommendations were inaccurate, including a recommendation to prescribe codeine to children despite a serious drug warning (black-box warning), and a different, too short, suggested post-operative follow-up.

**Fig. 7:**
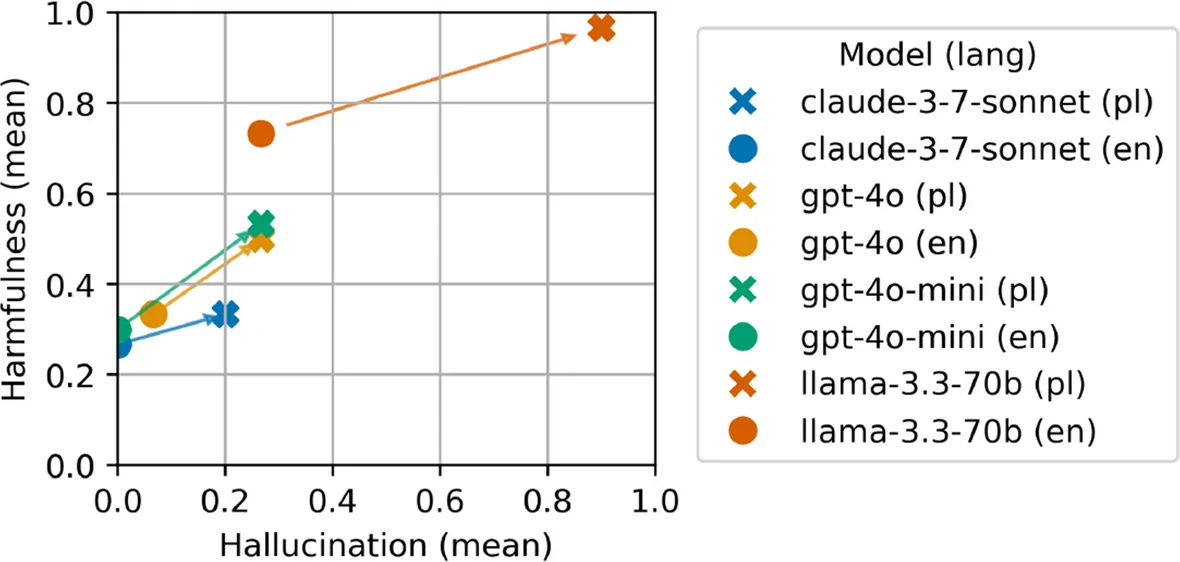
Mean harmfulness and hallucination scores for unprompted LLM-generated answers in Polish and English

An important observation can be made regarding the trained classifier using the Qwen3-Embedding-0.6B model coupled with the Support Vector Machine. It was shown that despite the Qwen3 model being declared multilingual, the classifier performs notably worse on Polish questions than on English. Similar behavior was observed in LLM-generated answers, as shown in Fig. 7.

The observed harmfulness rate was substantially higher in Polish questions. While explaining the reasons behind this difference was not the focus of the present study, language-specific performance gaps are well documented in existing benchmarks, as models trained explicitly on Polish corpora demonstrate superior results on language-specific tasks [36]. This is further supported by the present results, where bielik-v2.3-11b, despite being significantly smaller than llama-3.3-70b, demonstrated lower harmfulness on Polish questions. This suggests the importance of considering language when training LLMs, particularly if they are to be implemented in healthcare solutions as chatbots or classifiers.

An important finding of this study concerns the inter-rater reliability of hallucination labeling. The low Cohen’s kappa observed for hallucination (*κ = 0.387*) suggests that this construct is inherently subjective, as even experienced clinicians do not consistently agree on what constitutes a hallucination in an LLM-generated response. This has a practical implication: systems designed to detect or filter LLM outputs based on hallucination alone may be unreliable, as the ground truth itself is ambiguous. The analyses involving hallucination should therefore be treated as exploratory, and further investigation is needed before any definitive conclusions can be drawn.

The questions used in this study are shared publicly as supplementary material to ensure reproducibility. However, this openness introduces a potential misuse risk: a model could be fine-tuned specifically on these questions to produce safe-appearing responses without genuinely generalising to the broader space of patient queries. Practitioners deploying LLM-based systems in clinical or patient-facing settings are therefore advised to prepare their own held-out validation datasets, ideally constructed from questions representative of their specific patient population. This ensures that safety evaluations reflect real-world behaviour rather than performance on a known benchmark. Such an approach also has the advantage of being population-specific: a dataset drawn from the actual patients a system will serve is more likely to capture the language, concerns, and health-seeking patterns relevant to that deployment context.

## Conclusions

The results obtained in this study highlight the need to verify, tune, and develop responsible solutions when deploying the evaluated Large Language Models in dental care contexts. It was shown that the tested Large Language Models, when used directly and without guardrails, produced outputs that could pose potential safety risks to dental patients. A potential association between model hallucinations and harmfulness was observed; however, given the low inter-rater agreement on hallucination labeling, this relationship requires further investigation. Providing factual, reliable, and up-to-date knowledge bases that could be used with LLMs in RAG systems may nonetheless be a promising direction for reducing the harmfulness of the models. The results showed that the highest potential for harmful responses generated by LLMs was in the case of high-risk inquiry questions, such as home remedies for teeth whitening or diminishing tooth pain at home. In those cases, substances were suggested that could have a potentially harmful influence on tooth structure. In conclusion, the LLMs evaluated in this study can provide useful information on dental topics, but the results indicate that caution is warranted, particularly regarding suggestions for home treatment or substance use. These results suggest it is important to monitor contemporary publicly available language models in the context of their use by patients. It seems appropriate to increase public education about oral health. A potentially useful strategy for reducing the number of harmful responses is the use of safety-oriented prompts, which produced measurable results within the scope of this study. Future work could include testing languages other than Polish and English and their influence on the harmfulness of LLM-generated answers to patient questions, as well as further refinement and tuning of the harmfulness classification algorithms, including improved dataset preparation for both harmfulness assessment and classification processes. A limitation of this study is the relatively small number of total questions (*n=30*). This constraint may restrict the generalizability of the findings to the full complexity of real-world patient interactions. Furthermore, the purposive sampling approach, while grounded in clinical experience and forum review, may not fully capture the diversity of patient queries across different populations, languages, and healthcare settings. Consequently, this dataset should be interpreted as a targeted benchmark-style evaluation rather than an exhaustive assessment of real-world clinical diversity. An additional limitation concerns the low inter-rater agreement on hallucination labeling (*κ* = 0.387), which reduces confidence in conclusions drawn about the relationship between hallucination and harmfulness. Future studies should address this by involving a larger group of evaluators to establish more reliable ground-truth labels for hallucination. The results obtained in the study do not allow the definitive determination of whether the observed Polish-English differences arise from language-specific model training, model safety alignment policies, or other factors. Although translation errors are an unlikely source, as the Polish questions were prepared by a bilingual dentist and are available for verification in the supplementary materials, investigating the underlying causes of this difference was not an aim of the present study and remains an open question for future work. Finally, no correction for multiple comparisons was applied across the chi-squared analyses. While these were conducted as exploratory tests to characterize patterns in the data, this approach increases the risk of false-positive findings and the results should be interpreted with appropriate caution. As LLM outputs are stochastic and proprietary model behaviour may change silently with provider updates, the findings for proprietary models reflect responses generated between March and June 2025 and may not generalise to future versions. For open-source models, the exact version identifiers are provided in Supplementary Table S2, ensuring reproducibility.

## Supplementary Information

Supplementary Material 1

## Data Availability

All data generated or analyzed during this study are available from the corresponding author upon reasonable request.

## Abbreviations

AI: Artificial Intelligence
API: Application Programming Interface
CI: Confidence Interval
LLM: Large Language Model
NLP: Natural Language Processing
RAG: Retrieval-Augmented Generation
SVM: Support Vector Machine
